# Skeletal Muscle Gene Expression Signatures of Obese High and Low Responders to Endurance Exercise Training

**DOI:** 10.1210/clinem/dgad677

**Published:** 2023-11-21

**Authors:** Leona Kovac, Thomas Goj, Meriem Ouni, Martin Irmler, Markus Jähnert, Johannes Beckers, Martin Hrabé De Angelis, Andreas Peter, Anja Moller, Andreas L Birkenfeld, Cora Weigert, Annette Schürmann

**Affiliations:** Department of Experimental Diabetology, German Institute of Human Nutrition Potsdam-Rehbruecke (DIfE), Nuthetal 14558, Germany; German Center for Diabetes Research (DZD e.V.), München-Neuherberg 85764, Germany; Research Group Molecular and Clinical Life Science of Metabolic Diseases, Faculty of Health Sciences Brandenburg, University of Potsdam, Brandenburg 14469, Germany; German Center for Diabetes Research (DZD e.V.), München-Neuherberg 85764, Germany; Institute for Clinical Chemistry and Pathobiochemistry, Department for Diagnostic Laboratory Medicine, University Hospital Tübingen, Tübingen 72076, Germany; Department of Experimental Diabetology, German Institute of Human Nutrition Potsdam-Rehbruecke (DIfE), Nuthetal 14558, Germany; German Center for Diabetes Research (DZD e.V.), München-Neuherberg 85764, Germany; Institute of Experimental Genetics, Helmholtz Zentrum München, Neuherberg 85764, Germany; Department of Experimental Diabetology, German Institute of Human Nutrition Potsdam-Rehbruecke (DIfE), Nuthetal 14558, Germany; German Center for Diabetes Research (DZD e.V.), München-Neuherberg 85764, Germany; German Center for Diabetes Research (DZD e.V.), München-Neuherberg 85764, Germany; Institute of Experimental Genetics, Helmholtz Zentrum München, Neuherberg 85764, Germany; School of Life Sciences, Chair of Experimental Genetics, Technical University Munich, Freising 85764, Germany; German Center for Diabetes Research (DZD e.V.), München-Neuherberg 85764, Germany; Institute of Experimental Genetics, Helmholtz Zentrum München, Neuherberg 85764, Germany; School of Life Sciences, Chair of Experimental Genetics, Technical University Munich, Freising 85764, Germany; German Center for Diabetes Research (DZD e.V.), München-Neuherberg 85764, Germany; Institute for Clinical Chemistry and Pathobiochemistry, Department for Diagnostic Laboratory Medicine, University Hospital Tübingen, Tübingen 72076, Germany; Institute for Diabetes Research and Metabolic Diseases of the Helmholtz Zentrum München at the University of Tübingen, Tübingen 72076, Germany; German Center for Diabetes Research (DZD e.V.), München-Neuherberg 85764, Germany; Institute for Diabetes Research and Metabolic Diseases of the Helmholtz Zentrum München at the University of Tübingen, Tübingen 72076, Germany; Department of Internal Medicine IV, University Hospital Tübingen, Tübingen 72076, Germany; German Center for Diabetes Research (DZD e.V.), München-Neuherberg 85764, Germany; Institute for Diabetes Research and Metabolic Diseases of the Helmholtz Zentrum München at the University of Tübingen, Tübingen 72076, Germany; Department of Internal Medicine IV, University Hospital Tübingen, Tübingen 72076, Germany; German Center for Diabetes Research (DZD e.V.), München-Neuherberg 85764, Germany; Institute for Clinical Chemistry and Pathobiochemistry, Department for Diagnostic Laboratory Medicine, University Hospital Tübingen, Tübingen 72076, Germany; Institute for Diabetes Research and Metabolic Diseases of the Helmholtz Zentrum München at the University of Tübingen, Tübingen 72076, Germany; Department of Experimental Diabetology, German Institute of Human Nutrition Potsdam-Rehbruecke (DIfE), Nuthetal 14558, Germany; German Center for Diabetes Research (DZD e.V.), München-Neuherberg 85764, Germany; Institute of Nutritional Science, University of Potsdam, Nuthetal 14558, Germany

**Keywords:** obesity, exercise, responders, LASSO, gene expression, DNA methylation

## Abstract

**Context:**

Exercise training is known to improve glucose tolerance and reverse insulin resistance in people with obesity. However, some individuals fail to improve or even decline in their clinical traits following exercise intervention.

**Objective:**

This study focused on gene expression and DNA methylation signatures in skeletal muscle of low (LRE) and high responders (RES) to 8 weeks of supervised endurance training.

**Methods:**

We performed skeletal muscle gene expression and DNA methylation analyses in LRE and RES before and after exercise intervention. Additionally, we applied the least absolute shrinkage and selection operator (LASSO) approach to identify predictive marker genes of exercise outcome.

**Results:**

We show that the two groups differ markedly already before the intervention. RES were characterized by lower expression of genes involved in DNA replication and repair, and higher expression of extracellular matrix (ECM) components. The LASSO approach identified several novel candidates (eg, *ZCWPW2*, *FOXRED1*, *STK40*) that have not been previously described in the context of obesity and exercise response. Following the intervention, LRE reacted with expression changes of genes related to inflammation and apoptosis, RES with genes related to mitochondrial function. LRE exhibited significantly higher expression of ECM components compared to RES, suggesting improper remodeling and potential negative effects on insulin sensitivity. Between 45% and 70% of differences in gene expression could be linked to differences in DNA methylation.

**Conclusion:**

Together, our data offer an insight into molecular mechanisms underlying differences in response to exercise and provide potential novel markers for the success of intervention.

The number of people with overweight and obesity is increasing, with more than 1.9 billion adults affected worldwide (1). Obesity represents a major risk factor for multiple diseases, including cancer (2), cardiovascular disease (3), and type 2 diabetes (T2D) (4). Exercise training is able to improve glucose tolerance and reverse insulin resistance associated with obesity, accompanied by an increased lipid oxidation and mitochondrial biogenesis (5). However, the underlying molecular mechanisms responsible for high effectiveness of exercise interventions in improving whole-body insulin sensitivity remain largely unknown. Studies addressing human exercise are complicated by the existence of a subpopulation that does not benefit from exercise training in terms of improvement of body mass index (BMI), glycated hemoglobin A_1c_ (HbA_1c_) levels, and insulin sensitivity (6-8). In a previous publication, we identified low responders (LRE) and high responders (RES) to 8 weeks of endurance exercise, based on improvement in insulin sensitivity. The two subpopulations exhibited comparable increase in fitness, respiratory capacity, and abundance of tested mitochondrial enzymes in skeletal muscle after the training (6). Here, we performed skeletal muscle gene expression and DNA methylation analyses to identify molecular signatures of LRE and RES before and after exercise training. Additionally, least absolute shrinkage and selection operator (LASSO) regression was used to identify predictive markers of insulin sensitivity improvement.

## Materials and Methods

### Study Design

A subset of previously described participants, after exclusion of age outliers, was used (6). In brief, 18 individuals with overweight or obesity at high risk for developing T2D underwent 8 weeks of supervised endurance training. Participants performed 3 sessions/week, consisting of 30 minutes’ bicycle ergometer exercise and 30 minutes’ treadmill walking. Participants were asked not to change their dietary behavior (calorie intake and composition) during the intervention. Severe diseases (thyroid dysfunction, Cushing syndrome, acute and chronic infections, etc) were excluded by anamnesis including monitoring of routine laboratory parameters, electrocardiogram, and physical examination. Their clinical parameters were assessed 1 week before the start and 5 days after the end of intervention. Insulin sensitivity was determined by a 75-g oral glucose tolerance test (OGTT) and calculated by the method described by Matsuda and DeFronzo (9). All individuals provided detailed, informed written consent; the study protocol (NCT03151590) was approved by the ethics committee of the University of Tübingen and was in accordance with the Declaration of Helsinki.

### Gene Expression Analysis

Skeletal muscle biopsies were obtained from the lateral part of vastus lateralis muscle using a Bergström needle as previously described (10). Biopsies were taken 8 days before (baseline) and 5 days after the intervention in a resting state 60 minutes after the end of an OGTT. Gene expression was performed as previously described (10). In brief, total RNA was isolated from vastus lateralis biopsies using a miRNeasy Kit (Qiagen). Gene expression levels were determined using the Human Clariom S array (Thermo Fisher Scientific). Transcriptome Analysis Console (TAC; version 4.0.0.25; Thermo Fisher) was used for quality control and to obtain annotated normalized SST-RMA (signal space transformation-robust multichip analysis) gene-level data. Data are available from the GEO database at NCBI with the accession numbers GSE161749 (10) and GSE161750 (10). As outlined in the study design, 25 samples from 7 individuals (50, 58, 61, 62, 66, 67, and 72) were not included in this analysis.

### DNA Methylation Analysis

Genomic DNA was isolated from vastus lateralis biopsies using the Invisorb Genomic DNA Kit II (STRATEC Molecular GmbH). DNA methylation levels, after bisulfite treatment, were determined by the Infinium MethylationEPIC BeadChip (Eurofins Genomics GmbH). The data were processed using R (v.4) packages “meffil” (v.1.3.1) and “ChAMP” (v. 2.24.0) as described earlier (11, 12). Raw data are available in the GEO database under accession number GSE244359 (13).

### Kyoto Encyclopedia of Genes and Genomes Pathway Enrichment

Pathway enrichment analysis was performed using the Kyoto Encyclopedia of Genes and Genomes (KEGG) via the publicly available Database for Annotation, Visualization and Integrated Discovery (DAVID) Functional Annotation Tool (14, 15). The following criteria were applied to the search: count greater than or equal to 5 genes per KEGG term, *P* less than or equal to .05.

### Least Absolute Shrinkage and Selection Operator Regression

LASSO regression was applied to genes differentially expressed between low responders and high responders at baseline to identify genes whose expression was predictive of Matsuda index fold change (FC) in response to 8 weeks of exercise. The prediction models were built by randomly allocating 50% (n = 9) of participants in each group to a training data set and 50% (n = 9) of participants to a test data set 1000 times, resulting in 1000 prediction models. R version 4.1.3 was used with R-package “glmnet” version 4.1. λ Was estimated using a 5-fold cross-validation. A model was considered successful if the predicted parameter value in the test set was within 10% range from the measured value in at least 50% of participants.

### Statistical Analysis

*P* values were calculated using a limma *t* test for both transcriptome and DNA methylation analyses. The R-package ComplexHeatmap (v2.4.3) was used to create heat maps.

## Results

### Differences in the Effect of Exercise on Insulin Sensitivity Are Accompanied by Distinct Changes in Transcriptome and Epigenome

**Figure 1.**
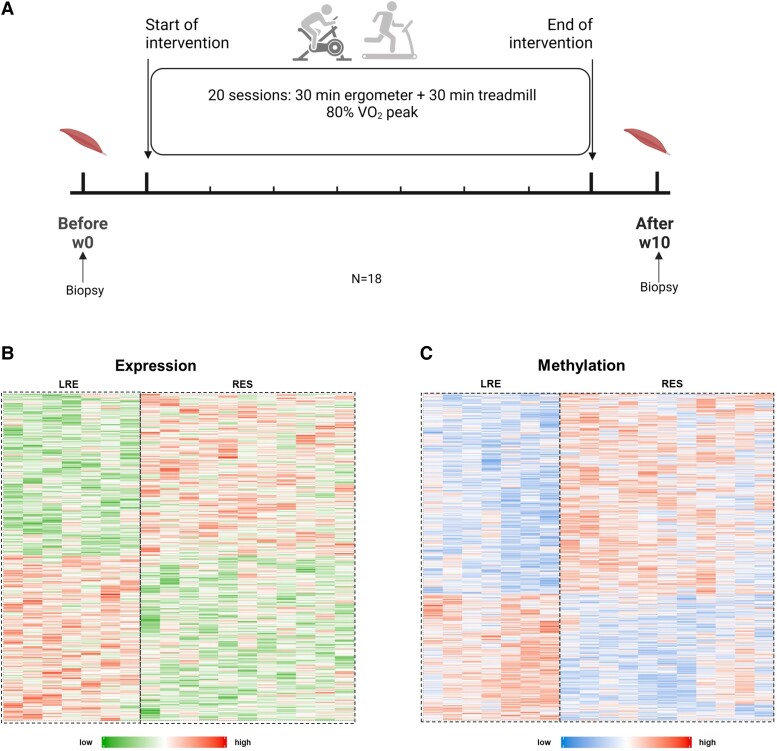
Low responders (LRE) and high responders (RES) exhibit distinct transcriptional and epigenetic adaptations to endurance exercise. A, Skeletal muscle biopsies were obtained from participants at rest, before and after exercise intervention. The heat maps depict the change in B, gene expression and C, DNA methylation levels between w0 and w10 in LRE and RES. Each row represents the ratio in expression of a gene or methylation level of a CpG site. B, Green and red represent decrease and increase in gene expression, respectively. C, Hypomethylation and hypermethylation are shown in blue and red, respectively. Figure created with BioRender.com.

To investigate the effect of exercise on skeletal muscle transcriptome and DNA methylome, a subgroup of a previously published cohort was used (6). Here, 18 (61% female) sedentary individuals with overweight or obesity underwent endurance exercise training (Fig. 1A). In accordance with previously published data, participants were categorized as LRE (N = 7) or RES (N = 11) based on their whole-body insulin sensitivity improvement, where RES had a 15% or greater increase in Matsuda index, while LRE exhibited a significant decline in insulin sensitivity after exercise training (Table 1). Baseline physical fitness as a surrogate of physical activity was not different between RES and LRE, and exercise intensity during intervention was adjusted to and controlled by the heart rate corresponding to 80% VO_2_peak. The heart rate was kept constant throughout the intervention, resulting in an increased exercise intensity during the intervention. The increase in exercise intensity was not different between LRE and RES (see Table 1). In addition to the different changes in glucose metabolism parameters, RES and LRE differed in their response in subcutaneous adipose tissue (AT) as reported recently (6). To test if LRE and RES react differently on the levels of gene expression and DNA methylation, we compared the degrees of changes in the skeletal muscle in response to the intervention. The heat maps shown in Fig. 1B and 1C demonstrate that the two groups can be distinguished by their alterations in gene expression and DNA methylation pattern. While sex-dependent effects cannot be entirely excluded, we did not observe separation of transcriptome nor methylome data based on sex (Supplementary Fig. S1) (16).

**Table 1.** Participant characteristics

Parameter	Pre		Post 8 wks		Fold change post/pre		*P* comparing fold change post/pre of RES and LRE	*P* comparing pre values of RES and LRE
Group	RES	LRE	RES	LRE	RES	LRE		
Sex	6 F, 5 M	5 F, 2 M						
Age, y	28.6 ± 4.72	27.6 ± 3.96						>.1
Height, cm	172 ± 10.3	171 ± 9.47	172 ± 10.3	171 ± 9.47				>.1
Body mass, kg	91.8 ± 17.1	96.9 ± 17.3	90.5 ± 17.3	96.7 ± 17.6	0.98 ± 0.02	1.00 ± 0.03	>.1	>.1
BMI	30.8 ± 3.65	33.3 ± 5.84	30.3 ± 3.81	33.3 ± 5.97	0.98 ± 0.02	1.00 ± 0.03	>.1	>.1
Waist to hip ratio	0.90 ± 0.05	0.87 ± 0.05	0.89 ± 0.05	0.87 ± 0.05	0.98 ± 0.02	1.00 ± 0.02	>.1	>.1
Total AT volume, L	39.0 ± 8.90	43.7 ± 16.3	37.8 ± 9.34	43.4 ± 15.8	0.96 ± 0.03	1.00 ± 0.05	>.1	>.1
Subcutaneous AT, L	14.7 ± 4.48	17.2 ± 7.88	13.7 ± 4.26	17.5 ± 8.07	0.93 ± 0.06	1.02 ± 0.06	.**015**	>.1
Visceral AT, L	3.54 ± 1.50	2.88 ± 1.32	3.33 ± 1.55	2.91 ± 1.13	0.93 ± 0.12	1.06 ± 0.15	.091	>.1
IAT_ergo_/BM, W/kg	1.08 ± 0.17	1.08 ± 0.28	1.34 ± 0.28	1.30 ± 0.31	1.23 ± 0.14	1.21 ± 0.10	>.1	>.1
VO_2_peak_ergo_/BM [mL/(kg*min)]	25.5 ± 2.95	23.1 ± 4.67	26.5 ± 4.15	26.5 ± 6.11	1.04 ± 0.13	1.15 ± 0.17	>.1	>.1
Glucose fasting, mmol/L	5.12 ± 0.40	4.97 ± 0.22	5.07 ± 0.40	4.87 ± 0.32	0.99 ± 0.05	0.98 ± 0.06	>.1	>.1
Glucose OGTT_120 min_, mmol/L	6.09 ± 1.06	5.32 ± 1.04	5.24 ± 0.88	6.10 ± 2.51	0.87 ± 0.10	1.18 ± 0.54	.085	>.1
Insulin fasting, pmol/L	114 ± 35.0	90.6 ± 45.5	93.0 ± 32.6	105 ± 31.6	0.84 ± 0.22	1.28 ± 0.32	.**013**	>.1
Insulin OGTT_120 min_, pmol/L	593 ± 345	458 ± 338	382 ± 294	578 ± 432	0.61 ± 0.22	1.44 ± 1.25	.**009**	>.1
ISI_Mats_	7.33 ± 3.09	10.9 ± 7.27	9.97 ± 4.52	8.67 ± 5.55	1.35 ± 0.25	0.82 ± 0.12	**<**.**001**	>.1
HbA_1c_, mmol/mol Hb	34.0 ± 2.86	34.0 ± 2.00	32.5 ± 3.03	34.1 ± 1.55	0.96 ± 0.07	1.01 ± 0.03	.0736	>.1
HbA_1c_,%	5.26 ± 0.26	5.26 ± 0.18	5.13 ± 0.28	5.27 ± 0.14	0.98 ± 0.04	1.00 ± 0.02	.0756	>.1
Leukocytes, 1/µL	7114 ± 1359	7130 ± 1475	6722 ± 1198	6801 ± 1683	0.96 ± 0.17	0.97 ± 0.22	>.1	>.1
CRP, mg/dL	0.53 ± 0.83	0.61 ± 0.66	0.38 ± 0.33	0.47 ± 0.53	1.33 ± 0.76	1.85 ± 2.10	>.1	>.1
Exercise performance, W	121 ± 35.1	113 ± 24.0	132 ± 27.5	123 ± 18.5	1.13 ± 0.19	1.11 ± 0.19	>.1	>.1

*P*-value<0.05 are shown in bold. **Abbreviations:** AT, adipose tissue; BMI, body mass index; CRP, C-reactive protein; F, female; HbA_1c_, glycated hemoglobin A_1c_; LRE, low responders; M, male; OGTT, oral glucose tolerance test; RES, high responders.

### Low Responders Exhibit Diminished Alterations of Gene Expression Related to Mitochondrial Metabolism Compared to High Responders

**Figure 2.**
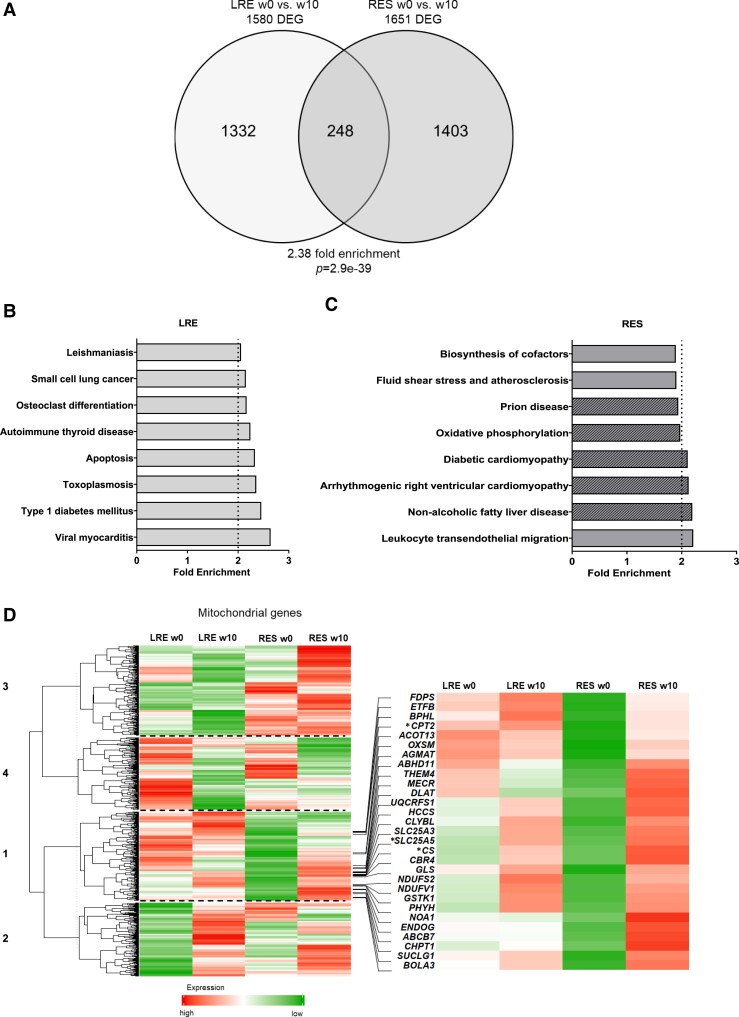
Differentially expressed genes in low responders (LRE) and high responders (RES) in response to 8 weeks of exercise. Venn diagram shows the numbers of differentially expressed genes (DEG) in LRE and RES, as well as the number of overlapping genes. A, Indicated is the fold enrichment and significance of the overlap, as calculated by a hypergeometric test. Bar plots represent the top unique enriched Kyoto Encyclopedia of Genes and Genomes pathways among genes affected in B, LRE and C, RES, ranked according to *P* value. Pathways containing genes related to mitochondrial function are marked with diagonal lines. The heat map shows expression levels of mitochondrial genes in LRE w0, LRE w10, RES w0, and RES w10, from left to right. Each row represents the mean expression of a gene; high and low expression are indicated in red and green, respectively. Signal intensity corresponds to the log-transformed magnitude of expression. D, Extracted genes are involved in metabolism and significantly altered exclusively in RES, asterisk marks examples from the text.

We have previously reported the skeletal muscle gene expression to long-term training in a different subset of the total cohort (10). Here, we expand the analysis specifically to LRE and RES and identified around 1600 differentially expressed genes (DEGs) (*P* ≤ .05) both in LRE and RES, with 248 common genes altered in both (Fig. 2A). The commonly affected genes were enriched only in the extracellular matrix (ECM)-receptor interaction process, in which 9 genes (*COL4A1*, *COL4A2*, *COL6A3*, *LAMA4*, *LAMB1*, *LAMC1*, *ITGA6*, *HSPG2*, and *THBS4*) were upregulated in response to exercise in both groups (Supplementary Fig. S2) (16). The DEGs in LRE and RES were enriched in 6 common pathways: ECM-receptor interaction, protein digestion and absorption, focal adhesion, PI3K-Akt signaling pathway, cell adhesion molecules, and pathways in cancer (data not shown). Pathways unique to LRE were related to immune reactions (eg, viral myocarditis, toxoplasmosis), platelet activation, and apoptosis (Fig. 2B). RES exhibited changes in genes enriched in mitochondria-related pathways (eg, oxidative phosphorylation, thermogenesis) and the calcium signaling pathway (Fig. 2C). This finding is consistent with our previous analyses in which we detected alterations in transforming growth factor beta (TGF-β) signaling, suggesting an increased inflammatory response in individuals who fail to improve insulin sensitivity in response to exercise (17).

Since mitochondrial function is vital to skeletal muscle function and response to exercise, we assessed the expression of mitochondrial genes in LRE and RES, before and after exercise intervention. Unsupervised clustering of the expression of mitochondrial genes (extracted from MitoCarta3.0 (18)) identified 4 clusters (Fig. 2D and Supplementary Table S1 (16)). The expression of genes in cluster 3 was different between LRE and RES at baseline, and remained largely unchanged in response to exercise in LRE and was partially upregulated in RES. Cluster 4 showed less pronounced differences at baseline and responded to intervention in both groups, however, to a lesser extent in RES. Clusters 1 and 2 contained genes mostly changed in RES and LRE, respectively (Fig. 2D). All clusters contained genes involved in a variety of mitochondrial processes (oxidative phosphorylation, mitochondrial dynamics, central dogma, protein import, and small-molecule transport). However, only cluster 1 comprised metabolism-related genes. Out of 139 mitochondria metabolism genes in cluster 1, 29 were differentially expressed between w0 and w10 in RES, while none were significantly altered in LRE. The metabolism genes affected exclusively in RES exhibited lower expression levels compared to LRE at baseline, and increased in response to exercise. Among these were *CS* (citrate synthase), *CPT2* (carnitine O-palmitoyltransferase 2), and *SLC25A5* (ADP/ATP translocase 2) (Fig. 2D, right panel with asterisk). Taken together, these data suggest that the LRE group shows diminished transcriptional adaptation of genes related to mitochondrial metabolism after exercise training.

### Identification of Genes Predictive of Insulin Sensitivity Improvement in Response to Exercise

**Figure 3.**
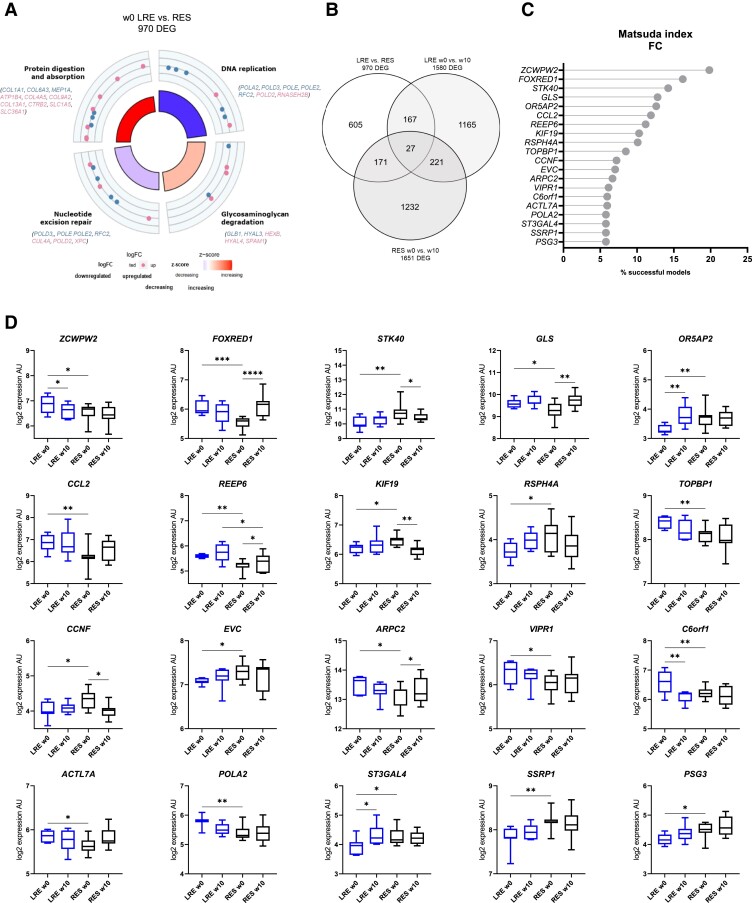
Identification of predictive markers for exercise intervention success. A, The circle plot shows the significantly enriched pathways among genes differentially expressed between low responders (LRE) and high responders (RES) at w0, including genes allocated to each pathway. Shown are logarithmic fold changes (logFC) (downregulated and upregulated in RES indicated in blue and pink, respectively) and *z* scores (from blue to red, summarizing decrease and increase in gene expression, respectively). B, Venn diagram depicts the proportion of differentially expressed genes (DEG) at baseline that are significantly altered in response to exercise training in LRE and RES. Top 20 genes predictive of Matsuda index FC as identified by LASSO regression are shown. C, The y-axis indicates the gene symbols and the x-axis the percentage of successful models which identified the gene as a predictive marker. Box plots display the expression levels of predictive markers identified with LASSO in LRE and RES. D, *P* values were determined using limma *t* test (*P < .05, **P < .01, ****P < .0001).

To investigate potential predictive markers for improved insulin sensitivity, we compared the LRE and RES skeletal muscle transcriptomes at baseline and identified 970 DEGs. These were enriched in DNA replication, glycosaminoglycan degradation, nucleotide excision repair, and protein digestion and absorption processes (Fig. 3A). RES showed lower messenger RNA levels of several DNA polymerase subunits (eg, *POLA2*, *POLD3*, *POLE2*). The glycosaminoglycan degradation and protein digestion and absorption pathways contained genes involved in ECM remodeling, the majority of which were expressed at a higher level in RES. To obtain an impression of how the DEGs at baseline change in the two groups after training, we overlapped the 970 genes with those affected by the exercise intervention of LRE and RES. Around 190 DEGs at baseline were significantly altered after exercise in each group, with only 27 changed in both (Fig. 3B). This indicates that the response to exercise in LRE and RES is mediated by distinct sets of genes.

Next, a LASSO machine-learning approach was applied to baseline DEGs to identify genes that may predict an improvement of insulin sensitivity in response to 8 weeks of exercise. A total of 555 successful models were generated, and the 20 most predictive genes were selected for further analysis (Fig. 3C). Of these, the top 3 genes encode a zinc finger histone methylation reader (*ZCWPW2*), mitochondrial respiratory chain complex I component (*FOXRED1*), and a putative regulator of nuclear factor-kappa-B and p53–mediated gene transcription (*STK40*) (19). Expression of 6 out of the 20 genes (*FOXRED1*, *STK40*, *GLS*, *KIF19*, *CCNF*, and *ARPC2*) were exclusively changed in RES, and 3 genes were altered in LRE (*OR5AP2*, *C6orf1*, and *ST3GAL4*); only *REEP6*, encoding receptor expression-enhancing protein 6, remained significantly different between LRE and RES at w10 (Fig. 3D). Eleven of the predictive markers were not significantly altered by exercise in either of the groups (see Fig. 3D), implying that they do not mediate the response to exercise but rather serve as indicators of metabolic condition prior to the intervention.

**Figure 4.**
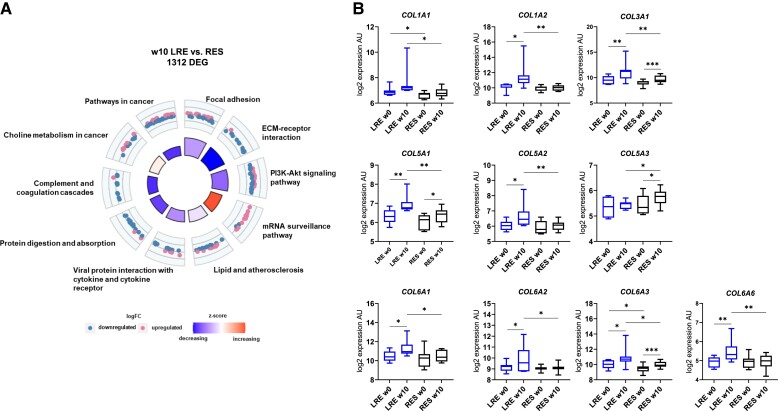
Low responders (LRE) and high responders (RES) are characterized by expression differences related to the extracellular matrix (ECM) at w10. The circle plot shows enriched pathways among genes differentially expressed between LRE and RES at w10. A, Shown are logFC (downregulated and upregulated in RES indicated in blue and pink, respectively) and *z* scores (from blue to red, representing decrease and increase, respectively). B, Box plots represent the expression levels of collagens differentially expressed between LRE and RES at w10. *P* values were determined using limma *t* test (*P < .05, **P < .01).

Another approach to understand why LRE failed to improve the metabolic health after intervention is to investigate the transcriptional differences between LRE and RES at w10. We detected 1312 DEGs between LRE and RES, enriched in pathways related to, among others, focal adhesion (*LAMA3*, *PDGFA*, *VEGFA*), ECM-receptor interaction (*COL1A1*, *ITGA5*, *LAMA3*), PI3K-Akt signaling (*IGF1R*, *PIK3R3*, *EFNA5*), protein digestion and absorption (*COL1A1*, *COL6A2*, *COL28A1*), and complement and coagulation cascades (*PROCR*, *F10*, *SERPINA5*) (Fig. 4A). The enriched pathways strongly suggested differences in ECM remodeling of the skeletal muscle in LRE and RES. Closer examination of the genes belonging to ECM-related pathways revealed a higher expression of collagens (Fig. 4B) and several other components and regulators of ECM (Supplementary Fig. S3 (16)) in LRE skeletal muscle after exercise training compared to RES. This observation supports former reports on induction of TGF-β signaling and consequent upregulation of collagens in LRE in response to 8 weeks of training (17, 20). Thus, higher expression of genes linked to ECM remodeling in LRE after exercise could be one factor among others leading to poor response to exercise in LRE group.

### DNA Methylation Potentially Regulates the Expression of Genes Involved in Mitochondrial Function and Extracellular Matrix Remodeling

**Figure 5.**
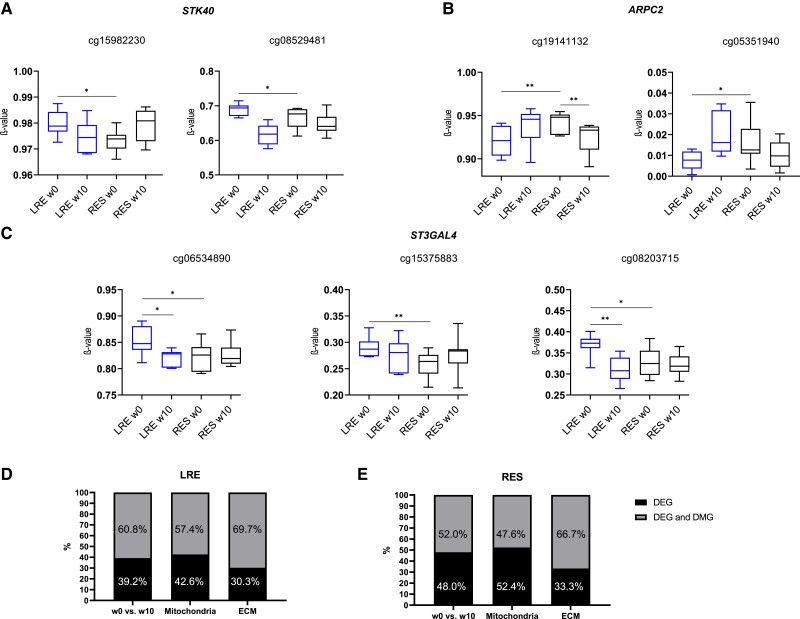
DNA methylation putatively regulates expression of genes involved skeletal muscle adaptation to endurance exercise. DNA methylation levels of CpG sites located in promoters of A, *STK40*; B, *ARPC2*; and C, *ST3GAL4* in low responders (LRE) and high responders (RES). Stacked bar charts summarize the proportion of differentially expressed genes (DEG) that may be affected by DNA methylation; all genes altered between w0 and w10 (left), mitochondrial genes altered between w0 and w10 (middle) and extracellular matrix (ECM)-related genes altered between w0 and w10 (right); in D, LRE, and E, RES. In gray, genes putatively regulated by DNA methylation, and in black genes without underlying changes in DNA methylation.

As we observed distinct patterns in genome-wide DNA methylation of LRE and RES (Fig. 1C), we examined the extent to which DNA methylation may contribute to the observed differences in gene expression between the two groups. Approximately 45% of DEGs at baseline were putatively epigenetically regulated through DNA methylation. They were enriched in pathways of FoxO signaling, endocytosis, cellular senescence, immune response, glycosphingolipid biosynthesis, and in Hippo and TGF-β signaling (Supplementary Fig. S4 (16)). Three predictive markers of Matsuda index improvement, as identified with LASSO, had 2 or more CpG sites within promoter region differentially methylated at baseline between LRE and RES. Higher expression of *STK40* and *ST3GAL4* (Fig. 3D) in RES was associated with lower promoter methylation levels (Fig. 5A and 5C), while lower expression of *ARPC2* (Fig. 3D) was associated with higher promoter methylation in RES (Fig. 5B).

In total, expression of approximately 61% genes in LRE and 52% genes in RES were potentially epigenetically regulated in response to exercise (w0 vs w10). Furthermore, it appears that in particular genes related to ECM are regulated by changes in DNA methylation (70% in LRE, 67% in RES), followed by mitochondrial genes (57% and 47%) (Fig. 5D and 5E). Overall, the degree of DNA methylation changes is lower in RES compared to LRE.

## Discussion

As the skeletal muscle is a major contributor to whole-body glucose homeostasis and shows pronounced physiological and molecular adaptations in response to exercise, we studied its transcriptome and epigenome in LRE and RES before and after an 8-week endurance exercise intervention to estimate the differences contributing to differential outcomes. Our data demonstrated that (1) LRE and RES skeletal muscle gene expression and DNA methylation profiles differ markedly; (2) both groups exhibited expression changes after training, however, with a low overlap; (3) LRE react with expression changes of genes related to inflammation and apoptosis, RES with genes related to mitochondrial function; (4) several mitochondrial genes had lower expression levels in RES at baseline and showed a strong activation in response to the training, whereas corresponding genes were less regulated in LRE; and (5) 61% and 52% of all DEGs may be epigenetically regulated in LRE and RES, respectively. Finally, (6) we identified prediction markers for the success of training intervention, including *ZCWPW2* and *ST3GAL4*, which exhibit a marked expression difference between the groups at baseline.

Interestingly, expression profiles of LRE and RES exhibited strong differences already before the intervention indicating a varying baseline and capacity to respond to the training. RES exhibited lower expression of genes involved in DNA replication and repair, and higher expression of ECM components prior to exercise intervention. ECM plays a crucial role in many physiological processes, including skeletal muscle development, growth, repair, contraction, and force transmission (21). In fact, improper ECM remodeling, even though incompletely understood, has been implicated in the development of insulin resistance by increasing the physical barrier to insulin transport or through integrin signaling (22). While exercise affected the expression of genes involved in ECM remodeling in both groups, the effect was stronger in LRE, resulting in significantly higher expression of multiple collagens at w10 (see Fig. 4A and 4B). Beside collagens, several other components of the ECM were expressed at significantly higher levels in LRE, including integrin alpha-5 (*ITGA5*), chondroadherin (*CHAD*), and thrombospondin-2 (*THBS2*). ITGA5 is a receptor for fibronectin, fibrinogen, and multiple immune factors, while CHAD promotes attachment of fibroblasts (19). Potentially, this could indicate excessive collagen deposition and development of fibrosis in skeletal muscle of LRE after exercise intervention. A potential mechanism leading to increased expression of ECM genes is the upregulation of TGF-β signaling and its downstream targets reported in LRE, including inflammatory markers and collagens, which negatively correlated with the improvement of insulin sensitivity (17, 20). Furthermore, collagen is known to be metabolized by macrophages (23) and as LRE exhibited an enrichment in immune pathways in response to exercise (Fig. 2B), we examined the expression levels of macrophage markers. Several of those markers (*CCR5*, *CD14*, *CD163*) were expressed at higher levels in LRE at w10, compared to RES (Supplementary Fig. S5 (16)).

Mitochondrial gene expression is known to be affected by endurance exercise, and differences in expression between RES and have been reported (7, 17). Considering we previously did not detect differences in respiration capacity between LRE and RES (6) but observed an impaired upregulation of some mitochondrial genes in LRE (17), we focused on alterations of transcripts related to mitochondrial function. Indeed, we identified a group of mitochondrial genes involved in the regulation of metabolism that is robustly induced only in RES. It included genes involved in mitochondrial fatty acid synthesis (*CBR4*, *MECR*, *OXSM*), in glutathione metabolism (*ABCB7*, *GSTK1*), and those encoding different transporters (*SLC25A3*, *SLC25A5*). Therefore, we hypothesize that LRE and RES differ in substrate transport into mitochondria, mitochondrial metabolism, and reactive oxygen species detoxification. In a study comparing lean and obese females with polycystic ovary syndrome, it was shown that the whole-body and skeletal muscle mitochondrial oxygen consumption were not different. However, obese females exhibited lower mitochondrial coupling and higher mitochondrial hydrogen peroxide emissions (24).

An important new finding of our study is the identification of marker genes predictive of improving insulin sensitivity in response to exercise. The top predictors *ZCWPW2*, *FOXRED1*, and *STK40*, identified by a machine-learning approach, the LASSO regression, have not yet been described in the context of insulin sensitivity and hence require further investigation. All 3 genes encode important proteins of general function. *ZCWPW2* is a paralog of *ZCWPW1*, a molecular partner of PRDM9 that was recently shown to facilitate double-strand break repair (25). *FOXRED1* is one assembly factor of complex I in the mitochondrial respiratory chain and plays an important role in mitochondrial function (26)*. STK40* encodes a serine/threonine-protein kinase and is supposed to be a negative regulator of nuclear-kappa-B and p53-mediated gene transcription (19). The majority of identified baseline marker genes were no longer different between LRE and RES at w10, suggesting these genes to be indicative of a metabolic state prior to intervention rather than mediators of exercise outcome. *REEP6* was the only putative marker of exercise response differentially expressed between LRE and RES at both time points investigated (Fig. 3D). Receptor expression-enhancing proteins (REEPs) are known to be involved in numerous physiological processes, including endoplasmic reticulum remodeling and trafficking of G protein–coupled receptors (27). *Reep6* knockout in mice results in reduced protein kinase A–mediated signaling and mitochondrial mass in brown AT, as well as exacerbated high-fat diet–induced insulin resistance and inflammation in AT, suggesting a role in metabolism regulation (28). While *REEP6* expression in AT is known to be reduced in people with T2D (28), studies investigating its role in skeletal muscle are lacking. Our data indicate a correlation between increased skeletal muscle *REEP6* expression and diminished response to exercise in people with overweight and obesity. Identification of exercise response markers would allow for a more personalized and effective approach to the treatment of obesity.

Taken together, the data presented offer more detailed insight into potential mechanisms underlying differential responses to exercise training. Even though the participants were given dietary questionnaires, they were unusable due to incompleteness, presenting a potential limitation to the study. Due to a small sample size and the descriptive nature of the study, more mechanistic approaches need to be applied to test suggested hypotheses. Furthermore, additional studies should be performed to determine whether LRE require a longer intervention time to achieve improvement or whether they profit more from a different type of exercise.

## Data Availability

Expression data presented in the current manuscript are either available in the supplementary data (16) or in publicly available repository GEO under accession numbers GSE161749 (10) and GSE161750 (10). Raw DNA methylation data are available under accession number GSE244359 (13).

## Clinical Trial Information

Clinical registration number NCT03151590 (registered January 9, 2017).

## Abbreviations

AT: adipose tissue
BMI: body mass index
CRP: C-reactive protein
DEG: differentially expressed gene
ECM: extracellular matrix
FC: fold change
HbA_1c_: glycated hemoglobin A_1c_
KEGG: Kyoto Encyclopedia of Genes and Genomes
LASSO: least absolute shrinkage and selection operator
LRE: low responders
OGTT: oral glucose tolerance test
REEP: receptor expression-enhancing protein
RES: high responders
T2D: type 2 diabetes
TGF-β: transforming growth factor beta
